# Emerging therapies in acute ischemic stroke

**DOI:** 10.12688/f1000research.21100.1

**Published:** 2020-06-05

**Authors:** Nicholas Liaw, David Liebeskind

**Affiliations:** 1 Department of Vascular Neurology, University of California, Los Angeles, 635 Charles E Young Drive South, Suite 225, Los Angeles, California, 90095-7334, USA

**Keywords:** stroke, acute ischemic stroke, thrombolytics, tPA, thrombectomy, stent-retriever, neuroprotection, collaterals, perfusion, telestroke, artificial intelligence, mobile stroke unit

## Abstract

Thrombolysis and mechanical thrombectomy have revolutionized the care of patients with acute ischemic stroke. The number of patients who can benefit from these treatments continues to increase as new studies demonstrate that not just time since stroke onset but also collateral circulation influences outcome. Technologies such as telestroke, mobile stroke units, and artificial intelligence are playing an increasing role in identifying and treating stroke. Stroke-systems-of-care models continue to streamline the delivery of definitive revascularization in the age of mechanical thrombectomy.

## Introduction

As recently as 25 years ago, all that was available to treat acute ischemic stroke was therapy to temporize the devastating sequelae of irreversibly injured brain tissue. The establishment of thrombolytic therapy and subsequent mechanical thrombectomy (MT) has revolutionized ischemic stroke therapy, allowing mitigation of ischemic stroke and at times outright complete recovery.

The goal of this article is to review advances in the diagnosis and treatment of ischemic stroke within the last 2 to 4 years. With the efficacy of thrombolytics and MT now well established, recent studies have pushed the boundaries of the type of patients who qualify for treatment and the window of opportunity for the efficacy of therapies after stroke onset. New imaging and selection technologies are being harnessed to help better identify patients who will benefit, and new systems are being set up to optimize the delivery of care to patients who need it. Finally, there have been advances and refinements in therapies aimed at prevention of strokes and rehabilitation of patients already afflicted with this disorder.

## Basis of acute stroke treatment

Ischemic stroke results from a cerebral artery blockage that leads to a loss of oxygenation in downstream brain tissue, ultimately resulting in neuronal cell death and irreversible neurological deficit. If the blockage is removed before substantial tissue injury ensues, reperfusion of the ischemic tissue may reverse or offset such risk of injury. The timing of intervention is a critical factor: in general, as time progresses after a cerebral artery is blocked, more tissue undergoes irreversible cell death.

## Stratification by collateral status

Despite a similar degree of vessel occlusion or the same degree of recanalization with MT or tissue plasminogen activator (tPA), patient outcomes can be significantly varied. The degree of collateral perfusion, mediated by arteries from adjacent vascular territories, determines the duration or time interval that ischemic tissue may survive before cell death ensues. Although it remains true in general that the sooner the intervention is given after stroke onset, the better the outcome ^1^, patients with better collaterals may benefit more from treatment when others with poor collaterals may not.

Collateral status can be determined via imaging, in which digital subtraction angiography (DSA) is the reference standard, delineating the time course or temporal features of the arterial, capillary, and venous phases of blood flow through the brain. Currently, computed tomographic perfusion (CTP) or magnetic resonance perfusion (MRP) is often used as a faster and less invasive alternative. Ischemic core (tissue that is irreversibly infarcted) and ischemic penumbra (tissue that is hypo-perfused and dependent on collaterals) can be inferred from the perfusion scans.

Using the RAPID automated software, the DEFUSE trials defined and validated cutoffs for penumbra as time to maximum of residual function (T _max_) of more than 6 seconds and core as the volume of brain where the magnetic resonance imaging (MRI) apparent diffusion coefficient was less than 600 × 10 ^−6^ mm ^2^/s ^2, 3^.

With CTP, mean transit time (MTT), cerebral blood flow (CBF), and cerebral blood volume (CBV) are calculated. Increased MTT with preserved or increased CBV suggests penumbra, whereas increased MMT with decreased CBV and decreased CBF is consistent with core ^4^.

A volume mismatch of the core and penumbra suggests salvageable tissue and relatively robust collateral support. Studies demonstrate that favorable collaterals slow the progression of penumbra to core ^5^ and that patients with better collaterals have better clinical outcome following intervention ^6^.

## Stroke imaging

The accurate selection of a treatable acute ischemic stroke is as important as the treatment itself. The current mainstays of evaluation include imaging technology, including computed tomography (CT)/CT angiography, MRI/magnetic resonance angiography (MRA), and DSA. CTP and MRP have become increasingly common. Commercial software such as RAPID (iSchemaView), Olea Sphere (Olea Medical), and Contact (Viz.ai) is now widely available and is able to process perfusion imaging for quick interpretation. RAPID in particular was used effectively to identify candidates for MT in the 2015 stent-retriever trials.

Recent years have seen an explosion of digital data. The emerging era of big data in stroke has led to the integration of artificial intelligence–based techniques such as deep learning and other forms of machine learning in which automated algorithms use large volumes of data to predict outcomes. Algorithms have been designed to identify and quantify the presence of acute stroke and vascular changes by using CT ^7^ and MRI ^8^ scans and have demonstrated non-inferiority compared with stroke experts. Clinical decision support software already available on the market uses artificial intelligence. Contact (Viz.ai), software that identifies large-vessel occlusions by using artificial intelligence, recently received US Food and Drug Administration approval ^9^.

## Thrombolysis

In 1995, the National Institute of Neurological Disorders and Stroke published a landmark study establishing the efficacy of the use of tPA ^10^. Since that time, additional studies have expanded inclusion criteria for tPA use up to 4.5 hours after symptom onset ^11^. There is demonstrated benefit across varying age, ischemic stroke type, and stroke severity.

However, tPA does carry a risk of hemorrhage and therefore its use in patients with a mild National Institutes of Health Stroke Scale (NIHSS) score remains ill defined. A recent study did not show benefit in providing treatment to stroke patients with an NIHSS score of less than 5, compared with aspirin therapy alone, but this study was terminated prematurely because of low trial recruitment ^12^. Other studies have explored further expanding the tPA time window on the basis of evidence of good collateral markers with moderate success. The WAKE-UP study selected “wake-up” strokes (where exact time of stroke onset is unknown, as the patient was asleep when the stroke occurred) with MRI acute diffusion restriction without more chronic changes on fluid-attenuated inversion recovery (FLAIR) sequences. tPA treatment in this population improved 90-day functional outcome but had increased symptomatic hemorrhagic transformation (HT) ^13^. In another study, collateral perfusion was assessed with either MRP or CTP, and patients with perfusion mismatch received tPA in an extended window up to 9 hours after symptom onset ^14^. A higher percentage of patients in the treatment arm had mild to no deficits but also had increased symptomatic HT ^14^.

The risk of HT after tPA has also been an impetus to find alternative pharmacologic thrombolytic agents. The most promising of these is tenecteplase, which is designed to have higher specificity than fibrin and has a longer half-life than tPA. NOR-TEST, a phase III clinical trial published 2017, demonstrated non-inferiority to tPA ^15^, and a meta-analysis of the tenecteplase clinical trials shows a lower risk of HT in the serious stroke subgroup at baseline ^16^. An ongoing phase III trial (TIMELESS) is investigating the efficacy of tenecteplase in an extended time window from 4.5 to 24 hours ^17^.

## Mechanical thrombectomy

Although tPA has been shown to be effective, its effectiveness may diminish with more proximal or larger occlusions ^18^. MT potentially solves this by physically removing the clot through endovascular approaches. Widespread acceptance of the efficacy of MT in treating acute stroke occurred in 2015 with the publication of a series of five clinical trials demonstrating benefit in MT up to 6 hours following stroke symptom onset using primarily second-generation stent-retriever devices ^19– 24^. More recently, the DAWN ^25^ and DEFUSE-3 ^2^ studies further expanded the therapeutic window for MT to up to 24 hours after symptom onset for patients with small core and large penumbra. These studies inform and shape the basis of the current standard of practice for MT.

There is a trend of studies that explore the expansion of treatment candidates for MT, just as there is for tPA. The landmark trials in 2018 limited inclusion to patients with small ischemic core and large penumbra (core of less than 70 mL in DEFUSE3 ^2^ and less than 51 mL in DAWN ^25^) but also with significant neurological deficit (NIHSS score of at least 6 in DEFUSE-3). In the recent SELECT study, a cohort prospective analysis of 105 patients showed that large core patients can also benefit from MT, with favorable outcome in patient with core sizes up to 100 mL ^26^. SELECT 2, a follow-up randomized clinical trial, is ongoing. Regarding MT in patients with mild stroke symptoms, a retrospective analysis and meta-analysis showed efficacy and safety similar to those of medical therapy alone ^27^.

Although the landmark MT trials focus on the efficacy of stent-retriever devices alone or in combination with an aspiration catheter, there is ongoing debate regarding the use of aspiration alone, which can make the procedure faster and less expensive ^28^. The COMPASS trial recently demonstrated that direct aspiration as first-pass thrombectomy (“ADAPT” technique) was non-inferior to using a stent-retriever device with respect to 90-day functional outcome ^29^.

New MT devices continue to be developed and undergo clinical trials. Notably, new stent-retriever devices are able to reach more distal vasculature (for example, M3 branches of the middle cerebral artery). Examples include the Tigertriever device (Rapid Medical), a stent-retriever that includes a slider to control the degree of stent expansion ^30^, and smaller versions of commercially available stent-retrievers, such as the Trevo XP ProVue 3×20 mm stent-retriever (“baby Trevo”) (Stryker) ^31^.

## Neuroprotective therapy

Current effective therapy for acute stroke focuses on removal of the arterial occlusion. Investigation into directly treating the ischemic brain tissue with pharmacologic and non-pharmacologic neuroprotective therapies has been ongoing since the 1990s but unfortunately has not yet yielded clear breakthroughs. Candidate agent mechanisms include excitotoxicity inhibitors, apoptosis inhibitors, free radical scavengers, and anti-inflammatory agents ^32, 33^.

Notably, however, the majority of neuroprotective clinical trials were performed prior to the thrombectomy era. Adjunct delivery of neuroprotective agents post- or peri-reperfusion is an unexplored and attractive possible therapy. Post hoc analyses have shown some neuroprotective agents to be more beneficial in the sub-population of patients who underwent MT, as seen in the uric acid trial ^34^. Nerinetide, an excitotoxic cell death pathway inhibitor, was recently tested in patients with MT in a large trial (ESCAPE NA1) ^35^. Although it failed to show a difference in 90-day functional outcome, the outcome was improved in nerinetide patients who did not receive tPA.

Some studies have explored tighter control of glucose ^36^ and blood pressure ^37^ immediately after acute stroke and in general have not shown these interventions to be beneficial. Regenerative medicine through stem cell therapy is an attractive concept that is pervasive throughout all of medicine. Trials investigating delivery of stem cells both acutely hours following ischemic stroke and chronically months to years after ischemic stroke are ongoing. Initial results show that therapy is safe but have yet to show functional efficacy ^38, 39^.

## Stroke care delivery

Delivery and access of acute stroke care are major issues regarding maximizing stroke treatment. Despite the benefit of acute intervention, only a small percentage of patients with stroke actually receive that intervention: with an estimated 691,000 acute ischemic strokes yearly in the US ^40^, only 13,000 MT were performed nationally as of 2016 ^41^ (less than 2%). This is due in part to system delays in arrival to and workflow at an intervention-capable center and to the lack of nearby intervention-capable centers (as in rural areas).

Different peri-hospital systems have been developed to quickly identify patients with acute stroke and decrease “door to needle” (DTN) (time from arrival to hospital to delivery of tPA) and “door to puncture” (time from arrival to hospital to groin access for MT) times. Policies such as pre-hospital notification, single call activation of the stroke team, moving patients to the scanner using the ambulance stretcher, and administering tPA in the scanner have helped to decrease DTN times. Individual centers have developed their own optimization schemes. The Helsinki University Central Hospital developed the seminal Helsinki protocol at their institution, leading to average DTN times of 20 minutes ^42^. There have been more systemic multi-hospital policy efforts as well. The American Heart Association/American Stroke Association developed the “Target: Stroke” initiative, leading to an improvement to DTN times of 59 minutes in participating hospitals ^43^.

Regarding geographical inaccessibility, solutions that have been developed include telestroke and mobile stroke units (MSUs). Current telestroke systems include a two-way video communication to allow a neurological exam to be conducted by the vascular neurologist and some method of remotely evaluating acute imaging. In this way, decision and treatment for tPA can be given prior to or without need for transfer from a remote hospital. Studies show improved DTN times in small hospitals with telestroke ^44^. In the event that MT or an otherwise higher level of care is indicated, the patient is transferred out (“drip and ship”). Frequently, multiple hospitals are funneled to a single transfer hospital, the so-called “hub and spoke” model. Alternatives include the “mothership” model, where hospitals without neurocritical care capability are bypassed in favor of a central hospital if acute stroke is suspected by emergency medical services (EMS). There is ongoing debate on which model leads to the best outcomes, DTN times, and general cost efficiency ^45^.

MSUs are ambulances that have been fitted with CT imaging technology and staffed with nurses, technicians, and physicians so that tPA can be delivered to the patient in the field after initial evaluation and imaging. In this way, the time spent during transfer by EMS to the hospital is eliminated, and decision making can be carried out directly by the stroke team. MSUs were first implemented in Germany in 2011 ^46^. Since that time, almost 20 locations have been using this technology worldwide. Reports from individual sites show improved time to tPA without decreasing safety ^46^, but the cost of creating and maintaining these units is high ^47^. The generalizability of MSUs remains to be fully determined.

## Conclusions

Our understanding of the mechanism and time course of acute ischemic stroke continues to advance. Now more than ever, ubiquitous digital connectivity and artificial intelligence technology are being leveraged to help treat this disorder. Recent years have seen a substantial increase in the fraction of patients whose acute ischemic stroke can be treated with tPA and MT and this population continues to expand. Although there have been advances in optimizing peri-hospital workflow and improving access to acute stroke therapy, more should be done to optimize care delivery, especially in rural areas and areas far from academic hospitals.

## Abbreviations

CBF, cerebral blood flow; CBV, cerebral blood volume; CT, computed tomography; CTP, computed tomographic perfusion; DSA, digital subtraction angiography; DTN, door to needle; EMS, emergency medical services; HT, hemorrhagic transformation; MRI, magnetic resonance imaging; MRP, magnetic resonance perfusion; MSU, mobile stroke unit; MT, mechanical thrombectomy; MTT, mean transit time; NIHSS, National Institutes of Health Stroke Scale; tPA, tissue plasminogen activator

## References

[1] Lees KR Bluhmki E von Kummer R: Time to Treatment With Intravenous Alteplase and Outcome in Stroke: An Updated Pooled Analysis of ECASS, ATLANTIS, NINDS, and EPITHET Trials. *Lancet.* 2010;375(9727):1695–703. 10.1016/S0140-6736(10)60491-6 20472172

[2] Albers GW Marks MP Kemp S: Thrombectomy for Stroke at 6 to 16 Hours With Selection by Perfusion Imaging. *N Engl J Med.* 2018;378(8):708–18. 10.1056/NEJMoa1713973 29364767 PMC6590673

[3] Straka M Albers GW Bammer R: Real-time Diffusion-Perfusion Mismatch Analysis in Acute Stroke. *J Magn Reson Imaging.* 2010;32(5):1024–37. 10.1002/jmri.22338 21031505 PMC2975404

[4] Austein F Riedel C Kerby T: Comparison of Perfusion CT Software to Predict the Final Infarct Volume After Thrombectomy. *Stroke.* 2016;47(9):2311–7. 10.1161/STROKEAHA.116.013147 27507864

[5] Sakoh M Østergaard L Røhl L: Relationship Between Residual Cerebral Blood Flow and Oxygen Metabolism as Predictive of Ischemic Tissue Viability: Sequential Multitracer Positron Emission Tomography Scanning of Middle Cerebral Artery Occlusion During the Critical First 6 Hours After Stroke in Pigs. *J Neurosurg.* 2000;93(4):647–57. 10.3171/jns.2000.93.4.0647 11014544

[6] Leng X Fang H Leung TWH: Impact of Collaterals on the Efficacy and Safety of Endovascular Treatment in Acute Ischaemic Stroke: A Systematic Review and Meta-Analysis. *J Neurol Neurosurg Psychiatry.* 2016;87(5):537–44. 10.1136/jnnp-2015-310965 26063928

[7] Nagel S Sinha D Day D: e-ASPECTS Software Is Non-Inferior to Neuroradiologists in Applying the ASPECT Score to Computed Tomography Scans of Acute Ischemic Stroke Patients. *Int J Stroke.* 2017;12(6):615–622. 10.1177/1747493016681020 27899743

[8] Boldsen JK Engedal TS Pedraza S: Better Diffusion Segmentation in Acute Ischemic Stroke Through Automatic Tree Learning Anomaly Segmentation. *Front Neuroinform.* 2018;12:21. 10.3389/fninf.2018.00021 29910721 PMC5996895

[9] FDA Approves Stroke-Detecting AI Software. *Nat Biotechnol.* 2018;36(4):290. 10.1038/nbt0418-290 29621226

[10] National Institute of Neurological Disorders and Stroke rt-PA Stroke Study Group: Tissue Plasminogen Activator for Acute Ischemic Stroke. *N Engl J Med.* 1995;333(24):1581–7. 10.1056/NEJM199512143332401 7477192

[11] Emberson J Lees KR Lyden P: Effect of treatment delay, age, and stroke severity on the effects of intravenous thrombolysis with alteplase for acute ischaemic stroke: A meta-analysis of individual patient data from randomised trials. *Lancet.* 2014;384(9958):1929–35. 10.1016/S0140-6736(14)60584-5 25106063 PMC4441266

[12] Khatri P Kleindorfer DO Devlin T: Effect of Alteplase vs Aspirin on Functional Outcome for Patients With Acute Ischemic Stroke and Minor Nondisabling Neurologic Deficits: The PRISMS Randomized Clinical Trial. *JAMA.* 2018;320(2):156–166. 10.1001/jama.2018.8496 29998337 PMC6583516

[13] Thomalla G Simonsen CZ Boutitie F: MRI-Guided Thrombolysis for Stroke with Unknown Time of Onset. *N Engl J Med.* 2018;379(7):611–622. 10.1056/NEJMoa1804355 29766770

[14] Ma H Campbell BCV Parsons MW: Thrombolysis Guided by Perfusion Imaging up to 9 Hours after Onset of Stroke. *N Engl J Med.* 2019;380(19):1795–1803. 10.1056/NEJMoa1813046 31067369

[15] Logallo N Novotny V Assmus J: Tenecteplase versus alteplase for management of acute ischaemic stroke (NOR-TEST): A phase 3, randomised, open-label, blinded endpoint trial. *Lancet Neurol.* 2017;16(10):781–788. 10.1016/S1474-4422(17)30253-3 28780236

[16] Xu N Chen Z Zhao C: Different doses of tenecteplase vs alteplase in thrombolysis therapy of acute ischemic stroke: Evidence from randomized controlled trials. *Drug Des Devel Ther.* 2018;12:2071–84. 10.2147/DDDT.S170803 30013325 PMC6038859

[17] https://clinicaltrials.gov/ct2/show/nct03785678 . Accessed October 19, 2019

[18] Del Zoppo GJ Poeck K Pessin MS: Recombinant tissue plasminogen activator in acute thrombotic and embolic stroke. *Ann Neurol.* 1992;32(1):78–86. 10.1002/ana.410320113 1642475

[19] Berkhemer OA Fransen PSS Beumer D: A randomized trial of intraarterial treatment for acute ischemic stroke. *N Engl J Med.* 2015;372(1):11–20. 10.1056/NEJMoa1411587 25517348

[20] Campbell BCV Mitchell PJ Kleinig TJ: Endovascular Therapy for Ischemic Stroke with Perfusion-Imaging Selection. *N Engl J Med.* 2015;372(11):1009–18. 10.1056/NEJMoa1414792 25671797

[21] Goyal M Demchuk AM Menon BK: Randomized Assessment of Rapid Endovascular Treatment of Ischemic Stroke. *N Engl J Med.* 2015;372(11):1019–30. 10.1056/NEJMoa1414905 25671798

[22] Jovin TG Chamorro A Cobo E: Thrombectomy within 8 Hours after Symptom Onset in Ischemic Stroke. *N Engl J Med.* 2015;372(24):2296–306. 10.1056/NEJMoa1503780 25882510

[23] Saver JL Goyal M Bonafe A: Stent-retriever thrombectomy after intravenous t-PA vs. t-PA alone in stroke. *N Engl J Med.* 2015;372(24):2285–95. 10.1056/NEJMoa1415061 25882376

[24] Goyal M Menon BK van Zwam WH: Endovascular thrombectomy after large-vessel ischaemic stroke: A meta-analysis of individual patient data from five randomised trials. *Lancet.* 2016;387(10029):1723–31. 10.1016/S0140-6736(16)00163-X 26898852

[25] Nogueira RG Jadhav AP Haussen DC: Thrombectomy 6 to 24 Hours after Stroke with a Mismatch between Deficit and Infarct. *N Engl J Med.* 2018;378(1):11–21. 10.1056/NEJMoa1706442 29129157

[26] Sarraj A Hassan AE Savitz S: Outcomes of Endovascular Thrombectomy vs Medical Management Alone in Patients With Large Ischemic Cores: A Secondary Analysis of the Optimizing Patient’s Selection for Endovascular Treatment in Acute Ischemic Stroke (SELECT) Study. *JAMA Neurol.* 2019. 10.1001/jamaneurol.2019.2109 31355873 PMC6664381

[27] Goyal N Tsivgoulis G Malhotra K: Medical Management vs Mechanical Thrombectomy for Mild Strokes: An International Multicenter Study and Systematic Review and Meta-analysis. *JAMA Neurol.* 2019;77(1):16–24. 10.1001/jamaneurol.2019.3112 31545353 PMC6763987

[28] Comai A Haglmüller T Ferro F: Sequential endovascular thrombectomy approach (SETA) to acute ischemic stroke: Preliminary single-centre results and cost analysis. *Radiol Med.* 2015;120(7):655–61. 10.1007/s11547-015-0501-9 25652155

[29] Turk AS Siddiqui A Fifi JT: Aspiration thrombectomy versus stent retriever thrombectomy as first-line approach for large vessel occlusion (COMPASS): A multicentre, randomised, open label, blinded outcome, non-inferiority trial. *Lancet.* 2019;393(10175):998–1008. 10.1016/S0140-6736(19)30297-1 30860055

[30] Kara B Selcuk HH Erbahceci Salik A: Single-center experience with the Tigertriever device for the recanalization of large vessel occlusions in acute ischemic stroke. *J Neurointerv Surg.* 2019;11(5):455–9. 10.1136/neurintsurg-2018-014196 30262656

[31] Haussen DC Lima A Nogueira RG: The Trevo XP 3×20 mm retriever ('Baby Trevo') for the treatment of distal intracranial occlusions. *J Neurointerv Surg.* 2016;8(3):295–9. 10.1136/neurintsurg-2014-011613 25948592

[32] Grupke S Hall J Dobbs M: Understanding history, and not repeating it. Neuroprotection for acute ischemic stroke: From review to preview. *Clin Neurol Neurosurg.* 2015;129:1–9. 10.1016/j.clineuro.2014.11.013 25497127

[33] Patel RAG McMullen PW: Neuroprotection in the Treatment of Acute Ischemic Stroke. *Prog Cardiovasc Dis.* 2017;59(6):542–548. 10.1016/j.pcad.2017.04.005 28465001

[34] Chamorro Á Amaro S Castellanos M: Uric acid therapy improves the outcomes of stroke patients treated with intravenous tissue plasminogen activator and mechanical thrombectomy. *Int J Stroke.* 2017;12(4):377–382. 10.1177/1747493016684354 28345429

[35] Hill MD Goyal M Menon BK: Efficacy and safety of nerinetide for the treatment of acute ischaemic stroke (ESCAPE-NA1): a multicentre, double-blind, randomised controlled trial. *Lancet.* 2020;395(10227):878–87. 10.1016/S0140-6736(20)30258-0 32087818

[36] Johnston KC Bruno A Pauls Q: Intensive vs Standard Treatment of Hyperglycemia and Functional Outcome in Patients With Acute Ischemic Stroke: The SHINE Randomized Clinical Trial. *JAMA.* 2019;322(4):326–335. 10.1001/jama.2019.9346 31334795 PMC6652154

[37] Anderson CS Huang Y Lindley RI: Intensive blood pressure reduction with intravenous thrombolysis therapy for acute ischaemic stroke (ENCHANTED): An international, randomised, open-label, blinded-endpoint, phase 3 trial. *Lancet.* 2019;393(10174):877–888. 10.1016/S0140-6736(19)30038-8 30739745

[38] Hess DC Wechsler LR Clark WM: Safety and efficacy of multipotent adult progenitor cells in acute ischaemic stroke (MASTERS): A randomised, double-blind, placebo-controlled, phase 2 trial. *Lancet Neurol.* 2017;16(5):360–368. 10.1016/S1474-4422(17)30046-7 28320635

[39] Kalladka D Sinden J Pollock K: Human neural stem cells in patients with chronic ischaemic stroke (PISCES): A phase 1, first-in-man study. *Lancet.* 2016;388(10046):787–96. 10.1016/S0140-6736(16)30513-X 27497862

[40] Benjamin EJ Blaha MJ Chiuve SE: Heart Disease and Stroke Statistics-2017 Update: A Report From the American Heart Association. *Circulation.* 2017;135(10):e146-e603. 10.1161/CIR.0000000000000485 28122885 PMC5408160

[41] Saber H Navi BB Grotta JC: Real-World Treatment Trends in Endovascular Stroke Therapy. *Stroke.* 2019;50(3):683–9. 10.1161/STROKEAHA.118.023967 30726185 PMC6407696

[42] Meretoja A Strbian D Mustanoja S: Reducing in-hospital delay to 20 minutes in stroke thrombolysis. *Neurology.* 2012;79(4):306–13. 10.1212/WNL.0b013e31825d6011 22622858

[43] Ebinger M Winter B Wendt M: Effect of the use of ambulance-based thrombolysis on time to thrombolysis in acute ischemic stroke: a randomized clinical trial. *JAMA.* 2014;311(16):1622–31. 10.1001/jama.2014.2850 24756512

[44] Heffner DL Thirumala PD Pokharna P: Outcomes of Spoke-Retained Telestroke Patients Versus Hub-Treated Patients After Intravenous Thrombolysis: Telestroke Patient Outcomes After Thrombolysis. *Stroke.* 2015;46(11):3161–7. 10.1161/STROKEAHA.115.009980 26396027

[45] Ciccone A Berge E Fischer U: Systematic review of organizational models for intra-arterial treatment of acute ischemic stroke. *Int J Stroke.* 2019;14(1):12–22. 10.1177/1747493018806157 30303811

[46] Walter S Kostopoulos P Haass A: Diagnosis and treatment of patients with stroke in a mobile stroke unit versus in hospital: A randomised controlled trial. * Lancet Neurol.* 2012;11(5):397–404. 10.1016/S1474-4422(12)70057-1 22497929

[47] Dietrich M Walter S Ragoschke-Schumm A: Is prehospital treatment of acute stroke too expensive? An economic evaluation based on the first trial. *Cerebrovasc Dis.* 2014;38(6):457–63. 10.1159/000371427 25531507

